# Prognostic value of CAPZA1 overexpression in gastric cancer

**DOI:** 10.3892/ijo.2013.1867

**Published:** 2013-03-27

**Authors:** YOUNG-JOON LEE, SANG-HO JEONG, SOON-CHAN HONG, BOK-IM CHO, WOO-SONG HA, SOON-TAE PARK, SANG-KYUNG CHOI, EUN-JUNG JUNG, YOUNG-TAE JU, CHI-YOUNG JEONG, JAE WON KIM, CHANG WON LEE, JIYUN YOO, GYUNG HYUCK KO

**Affiliations:** 1 Department of Surgery, Postgraduate School of Medicine, Gyeongnam Regional Cancer Center, Gyeongsang National University, Jinju, Republic of Korea; 2 Department of Microbiology, Research Institute of Life Science, College of Natural Sciences, Gyeongsang National University, Jinju, Republic of Korea; 3 Department of Pathology, Gyeongsang National University, Jinju, Republic of Korea

**Keywords:** stomach neoplasm, CAPZA1, F-actin capping protein, immunohistochemistry, biomarker

## Abstract

F-actin capping protein α1 subunit (CAPZA1) was previously identified in a proteomic analysis of human gastric cancer clinical specimens and selected for further study. The association between CAPZA1 overexpression, detected by immunohistochemistry, and clinicopathological features including survival were evaluated. *In vitro* gain-of-function and loss-of-function approaches were utilized to assess the function of CPAZA1 in malignancy. Univariate analysis revealed that poorly differentiated disease, according to the World Health Organization (WHO) classification, advanced T stage, positive lymph nodes, high TNM stage, D2 lymph node dissection, adjuvant chemotherapy and CAPZA1 underexpression were significantly associated with cancer-related death (p<0.05); however, only high TNM stage remained significantly associated by multivariate analysis (p<0.01). CAPZA1 overexpression was associated with well differentiated histology, smaller tumor size, lower T stage, absence of lymph node metastasis, lower TNM stage, lower recurrence rate and longer survival time, compared to CAPZA1 underexpression. *In vitro*, forced expression of CAPZA1 caused a significant decrease in gastric cancer cell migration and invasion, whereas CAPZA1 depletion had the opposite effect. The present study suggests that CAPZA1 could be a marker of good prognosis in gastric cancer and shows that CAPZA1 is associated with decreased cancer cell migration and invasion.

## Introduction

Although the incidence and mortality of gastric cancer (GC) has gradually decreased in East Asia, the disease remains the second most frequent cause of death in Korea (1). GC is the fourth most common type of cancer and the second leading cause of death worldwide. Nearly one million new cases are diagnosed each year (2). Surgery is an effective treatment for GC, and recent research has shown that chemotherapy is an effective adjuvant therapy for East Asian patients following radical surgery (3).

Recent attempts have been made to identify biomarkers predicting survival or recurrence in GC. Human epidermal growth factor receptor 2 (Her2) is associated with aggressive behavior in 15–25% of breast cancer cases and approximately 10% of GC cases. Combined therapy with trastuzumab (a monoclonal antibody against HER2) and conventional chemotherapy is superior to conventional chemotherapy alone in the treatment of GC (4). Despite advances in the understanding of GC at the molecular level and the emergence of targeted therapy in GC, predictive biomarkers have remained elusive (5–7).

We previously conducted a proteomic analysis of 152 human gastric cancer clinical specimens to identify proteins differentially expressed in gastric tumor tissues compared to normal tissues. Out of 430 proteins, the analysis identified F-actin capping protein α1 subunit (CAPZA1) (8). Capping protein (CP) is a heterodimer composed of α and β subunits. Each subunit has a mass of ∼30 kDa. Lower organisms, including *Saccharomyces cerevisiae*, *Caenorhabditis elegans* and *Drosophila melanogaster*, have one gene and one isoform for each of the CP α and β subunits (9–11). Vertebrates have three α subunit isoforms encoded by three different genes. Two very similar isoforms for the CP α subunit, α1 and α2, have been identified in chickens, mice and humans (12–14). The α3 subunit is only expressed in mouse testicular germ cells and has a different amino acid sequence than α1 and α2 (15). In contrast, three β subunit isoforms (β1, β2 and β3) are produced from one gene by alternative splicing (16–18).

CP has been identified in platelets and its interaction with cytoskeletal actin has been characterized. One study (19) reported CP release from activated platelet cytoskeleton 5 to 15 sec following activation with thrombin; however, another study reported that CP plays an important role in maintaining the resting form of platelets by binding to available barbed ends (20).

The aim of the present study was to determine whether CAPZA1 may be used as a prognostic marker in GC. The potential association between CAPZA1 overexpression, assessed by immunohistochemistry and clinicopathological features, including survival, was evaluated. Results showed that CAPZA1 overexpression in GC is associated with well differentiated histology, smaller tumor size, lower T stage, absence of lymph node (LN) metastasis, lower TNM stage, lower recurrence rate and longer survival time, compared to CAPZA1 underexpression.

## Materials and methods

### Stomach tissue samples

GC tissue specimens were surgically resected from 327 patients who underwent gastrectomy at Gyeongsang National University Hospital between January 1, 2004 and December 31, 2007. Medical charts and pathological reports were reviewed to assess clinicopathological parameters such as age, gender, histological subtype, presence of lymphatic invasion, invasion depth, presence of LN or distant metastasis and pathological TNM stage (AJCC, 7th edition). In cases of death, the cause of death was identified by consulting the National Statistical Office of the Republic of Korea. Among the 327 patients, 98.8% had undergone curative resection (R0) according to the guidelines of the International Union Against Cancer. Clinical outcome was evaluated during the time period extending from the date of surgery to death or January 31, 2010. Cases lost to follow-up and non-GC-related deaths were regarded as censored data in the survival analysis. The study was approved by the Institutional Review Board of Gyeongsang National University Hospital (GNUHIRB 2009-54).

### Tissue microarrays (TMA)

Core tissue biopsy specimens (2 mm in diameter) were obtained from individual formalin-fixed and paraffin-embedded archival tissue (donor blocks). These were arranged in new recipient paraffin blocks using a trephine apparatus (Quick-RAY™, Unitma, Seoul, Korea). One tissue core from the area near the invasive front was analyzed. TMA blocks were constructed for all 327 cases.

### Immunohistochemistry

**Figure 1:**
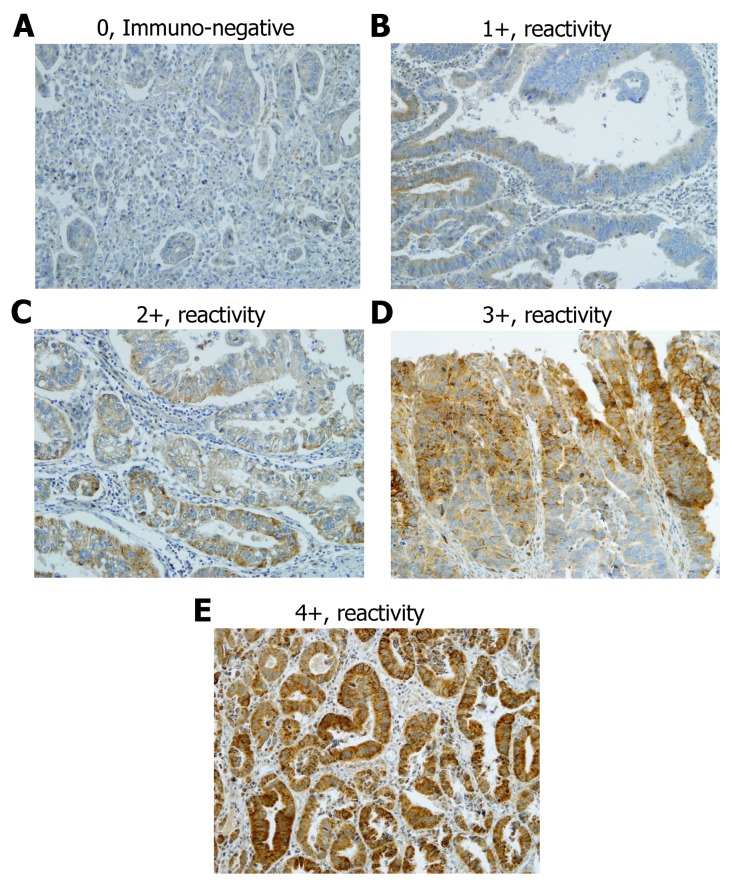
Immunohistochemical analyses of CAPZA1 expression in gastric carcinoma tissues. Cytoplasmic reactions were scored according to the percentage of CAPZA-1-positive cells as follows: (A) immunonegativity; (B) 1+ reactivity intensity (1–24%); (C) 2+ reactivity intensity (25–49%); (D) 3+ reactivity intensity (50–74%); (E) 4+ reactivity intensity (75–100%).

Immunohistochemical staining was performed using 4 *μ*m-thick tissue sections. Briefly, the tissue section was deparaffinized and rehydrated. To reduce non-specific background staining due to endogenous peroxidase, the slide was incubated in 3% H_2_O_2_ for 10 min. For epitope retrieval, the specimen was heated for 20 min in 10 mmol/l citrate buffer (pH 6.0) in a microwave oven (700 W). After incubation with Ultra V Block (Lab Vision Corporation, Fremont, CA, USA) for 7 min at room temperature to block background staining, the slide was incubated at room temperature for 1 h with a rabbit polyclonal antibody specific for CAPZ (ProteinTech Group, Chicago, IL, USA; dilution 1:50). Antibody binding was detected using the UltraVision LP detection system (Lab Vision Corporation) in accordance with the manufacturer’s recommendations. Color development was performed with 3-3′-diaminobenzidine and the slides were then counterstained with hematoxylin. The expression of CAPZ was scored by a pathologist who was blinded to the clinicopathological data. Cytoplasmic immunoreactivity was scored from 1 to 4 according to the percentage of cells positive for CAPZ: 1+ (1–24%), 2+ (25–49%), 3+ (50–74%) or 4+ (75–100%) (Fig. 1) (21).

### RNA interference

Two different siRNA oligonucleotide duplexes targeting CAPZA1 genes (designated siCAPZA1-A and siCAPZA1-B) were purchased from Samchully (Seoul, Korea). The sequences were 5′-CUG UGA AGA UAG AAG GAU A-3′ (siCAPZA1-A) and 5′-GGA ACA AGA UAC UCA GCU A-3′ (siCAPZA1-B). Transient transfection of each siRNA duplex was achieved using the siLentiFect™ Reagent (Bio-Rad) in accordance with the manufacturer’s instructions. After 24 h of incubation, the cells were harvested. The efficiency of each siRNA duplex was confirmed by western blot analysis using anti-CAPZA1 antibody.

### Construction of the CAPZA1 expression plasmid and transfection

Human CAPZA1 cDNA was amplified by polymerase chain reaction (PCR) using the following two sets of primers: 5′-AGCTAAGCTTCCACCATGGCCGACTTCGATGAT-3′ and 5′-AATTGAATTCTTAAGCATTCTGCATTTCTTT-3′. The PCR products were cloned into the expression vector, pCMV-Tag2B/G418 (Invitrogen). MKN-45 cells were transfected with CAPZA1-encoding plasmid using the TerboFect™ reagent (Fermentas, Glen Burnie, MD, USA) in accordance with the manufacturer’s instructions. After 48 h of incubation, the cells were exposed to neomycin for selection. CAPZA1 expression in neomycin-resistant clones was assessed by western blot analysis. The pCMV-Tag2B/G418 empty vector was used to generate control cells resistant to neomycin.

### Invasion and migration assays

Invasion across an ECMatrix-coated membrane was assayed using the QCM™ 24-Well Cell Invasion Assay (Millipore) (6). Migration across the membrane was assayed using the QCM 24-Well Cell Migration Assay (Millipore). Both assays were used in accordance with the manufacturer’s instructions. The following steps were applied to both assays. Cells were grown in 6-well plates to 70% confluence. Cells were serum-starved for 18 h. Serum-starved cells were harvested and resuspended in 1 ml of serum-free medium. A 250 *μ*l volume of cell suspension (1×10^6^ cells/ml) was added to each insert and 500 *μ*l of appropriate medium containing 20% FBS (chemoattractant) was added to the lower chamber. The chambers were incubated for 20 h at 37°C in a 5% CO_2_ atmosphere. All cells and medium remaining in the insert were removed by pipetting. The invasion chamber insert was transferred into a clean well containing 225 *μ*l of pre-warmed Cell Detachment Solution and incubated for 30 min at 37°C. The insert was then removed from the well. A 75 *μ*l volume of lysis buffer/dye solution (CyQuant GR Dye, 1:75 with 4X lysis buffer) was added to each well, which already contained 225 *μ*l of Cell Detachment Solution and the cells that invaded or migrated. After 15 min at room temperature, a 200 *μ*l volume of the mixture was transferred to a 96-well plate and fluorescence was assessed using a fluorescence plate reader and a 480/520 nm filter set.

### Proliferation assay

The cells were placed in a 24-well plate at a concentration of 5×10^5^ cells/well. After 1 to 4 days of incubation at 37°C in an atmosphere of 5% CO_2_, the cells were trypsinized and resuspended in 3 ml of appropriate medium. Cell suspensions were centrifuged at 1,000 rpm for 5 min. Cell pellets were resuspended in 1 ml of appropriate medium. The cells were stained with trypan blue and viable cells were counted using a hemocytometer.

### Statistical analysis

Statistical analysis was performed using the PASW Statistics 18.0 software (IBM Corporation, Somers, NY, USA). The data are presented as means ± SD. Significance was assessed using the χ^2^ test, Student’s t-test, binary logistic regression test, and the Kaplan-Meier method. All statistical tests were two-sided and a p-value of <0.05 was considered to be statistically significant.

## Results

### Patient demographics

The average age of the patients was 62.1 years. The male to female ratio was 1.8 to 1. The mean tumor size was 4.2±2.7 cm and the mean number of metastatic LN was 2.3±5.4. The number of tumors for each TNM stage was as follows: stage I, 69.4% (n=227); stage II, 13.1% (n=43); stage III, 13.5% (n=43); and stage IV, 4.0% (n=13). With regard to surgery type, subtotal, total and proximal gastrectomy were performed in 231, 74 and 22 patients, respectively. The LN dissections performed were D1+ (115, 35%) and D2 (212, 65%). The mean period of follow-up was 55.3±23.2 months. Recurrence occurred in 18.3% of the cases (n=60) and cancer-related deaths occurred in 15% of the cases (n=49) (Table I).

CAPZA1 protein expression was detected by immunohistochemistry (IHC) in all 327 GC tissue specimens. The intensity of CAPZ expression in the cytoplasm of cancer cells varied. Of the 327 cases, 17.4% cases (57) scored 0, 22.3% (73) scored 1+, 25.1% (82) scored 2+, 19.6% (64) scored 3+, and 4.0% (13) scored 4+. Scores of 0 and 1+ were considered negative for CAPZA1 protein overexpression and scores of 2+, 3+ and 4+ were considered positive. Normal and metaplastic epithelial cells, smooth muscle cells, vascular endothelial cells and plasma cells were weakly positive (Fig. 1).

### Univariate and multivariate analysis of risk factors for cancer-related death in GC

Differentiation was assessed according to the World Health Organization (WHO) classification. Univariate analysis revealed that poor differentiation (24.2%) was associated with cancer-related death to a greater extent than well differentiated histology (6.1%) or moderate differentiation (13.2%) (p= 0.04). Advanced T stage, high LN stage, high TNM stage, D2 lymph node dissection, adjuvant chemotherapy and CAPZA1 underexpression were significantly associated with cancer-related death (Table II, p<0.05); however, when a multivariate analysis was performed, only high TNM stage remained significantly associated with cancer-related death (p<0.01).

### CAPZA1 overexpression is associated with a lower rate of tumor invasion, LN metastasis and recurrence

Based on the immunohistochemical staining of CAPZA1 in TMAs, patients were divided into two groups: CAPZA1 overexpression (CAZA1-OE) and CAPZA1 underexpression (CAPZA1-UE). CAPZA1-OE was associated with well differentiated, moderately-differentiated or mucinous histology, according to the classification of the WHO (p<0.01); however, there was no statistically difference in terms of intestinal and diffuse types according to the Lauren classification (p=0.37). CAPZA1-OE correlated with smaller tumor size (3.7 cm) compared to CAPZA1-UE (4.8 cm) (p<0.01). CAPZA1-OE showed a significantly higher rate of T1- and T2-stage cancer (62.1 vs. 14.4%) than CAPZA1-UE (40.3 vs. 7.8%), and a lower rate of T3- and T4-stage cancer (41.9 vs. 15.9% and 10.1 vs. 7.7%, respectively) (p<0.01). In addition, the absence of LN metastasis in CAPZA1-OE (72.8%) was significantly higher than in CPAZA1-UE (56.6%) (p=0.01). In terms of TNM stage, the proportion of patients with TNM stage I in CAPZA1-OE was higher than that in CAPZA1-UE (70.8 vs. 44.2%); however, the proportion of patients with TNM stage II and III–IV in CAPZA1-UE was higher than that in CAPZA1-OE (II, 27.9 vs. 11.3%, III–IV, 27.9 vs. 17.9%). D2 LN dissection was higher in CAPZA1UE (73.6%) than CAPZA1OE (59.5%), and treatment with adjuvant chemotherapy was also higher in CAPZA1UE (65.9%) than CAPZA1OE (53.8%); however, the recurrence rate of CAPZA1-UE was significantly higher than that of CAPZA1-OE (25.6 vs. 13.8%), as was the rate of cancer-related death (21.7 vs. 10.8%) (Table III).

### CAPZA1 overexpression is associated with longer survival time than CAPZA1 underexpression

**Figure 2:**
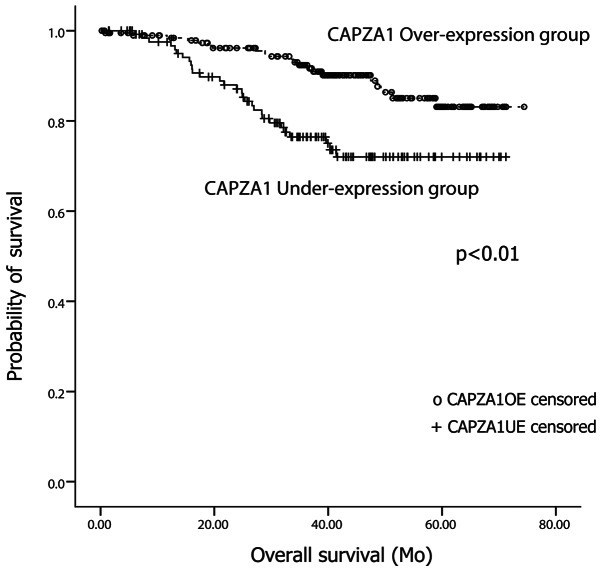
Kaplan-Meier survival analysis was then performed to compare the outcomes of patients overexpressing or under expressing CAPZA-1. Patients with CAPZA-1 overexpression (68±1.3 months) showed longer survival times than patients with CAPZA-1 underexpression (58±2.1 months). The difference between the two groups was significant (log-rank test, p<0.01).

Among the 327 patients, 77 (23.6%) have died, of whom, 50 died of cancer recurrence. A Kaplan-Meier survival analysis was performed to compare the outcome of patients in the CAPZA1-UE group to that of patients in the CAPZA1-OE group. Patients with CAPZA-1 overexpression showed a longer survival time (68±1.3 months) than patients with CAPZA-1 underexpression (58±2.1 months). The difference between the two groups was significant (log-rank test, p<0.01) (Fig. 2).

### CAPZA1 expression in GC cell lines

**Figure 3:**
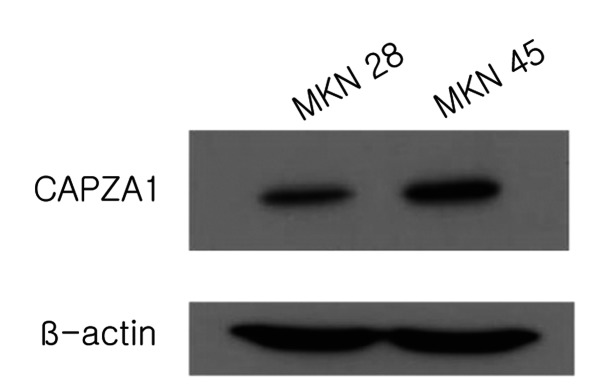
Upregulation of CAPZA1 expression in gastric cancer cell lines (MKN 28 and MKN 45).

To assess the mechanisms underlying the association of CAPZA1 with good prognosis, the expression of CAPZA1 protein was assessed in two different human GC cell lines. Interestingly, CAPZA1 overexpression was observed in MKN-45, a poorly invasive GC cell line, whereas CAPZA1 underexpression was observed in MKN-28, a highly invasive cell line (Fig. 3) (22).

### Depletion of CAPZA1 expression triggered GC cell migration and invasion in vitro

**Figure 4:**
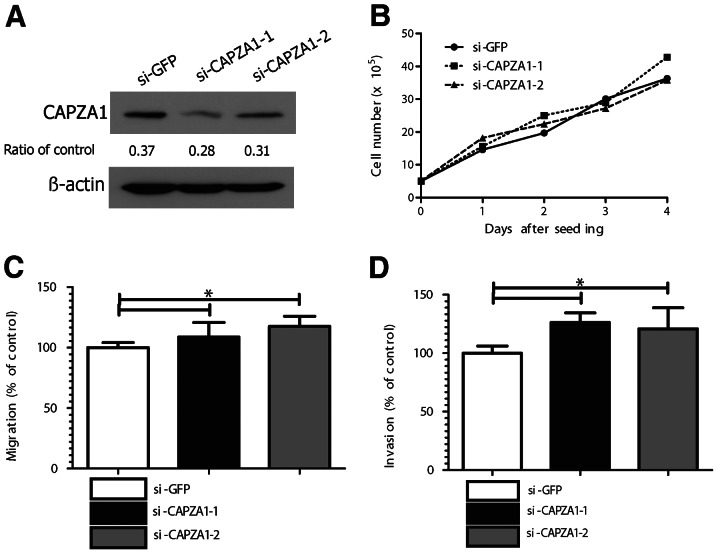
Depletion of CAPZA1 expression promotes gastric cancer cell migration and invasion. (A) The specificity of the two different CAPZA1 siRNAs in downregulating CAPZA1 gene expression. MKN 45 stably transfected with empty (si-GFP) or CAPZA1 si-RNAs (si-CAPZA1-1, si-CAPZA1-2) were analyzed by immunoblotting with an antibody to CAPZA1. An antibody to β-actin was used as an equal loading control. (B) The proliferation rates of the CAPZA1-depleting MKN 45 cells were not significantly different from those of control cells. (C) The effect of CAPZA1 depletion on *in vitro* migration ability of MKN 45 cells. The migration rates of CAPZA1 depleting MKN 45 cell lines were markedly increased than that of the control cells. (D) The effect of CAPZA-1 depletion on *in vitro* invasion ability of MKN 45 cells. The invasion rates of CAPZA1 depleting MKN 45 cell lines were markedly increased compared to that of the control cells. ^*^p<0.01.

The effect of CAPZA1 depletion on tumor cell proliferation, migration and invasion was assessed in MKN-45 cells using CAPZA1-siRNA. The specificity of the two different CAPZA1 siRNAs (si-CAPZA1-1, si-CAPZA1-2) was confirmed (Fig. 4A). The proliferation rate of CAPZA1-depleted MKN-45 cells was not different from that of control cells (Fig. 4B); however, the migration rate of CAPZA1-depleted MKN-45 cells (si-CAPZA1-1, si-CAPZA1-2) was markedly increased compared to that of control cells (p<0.05, Fig. 4C). The invasion rate of the CAPZA1-depleted MKN-45 cells was also markedly increased compared to that of control cells (p<0.01, Fig. 4D).

### CAPZA1 overexpression suppressed GC cell migration and invasion in vitro

**Figure 5:**
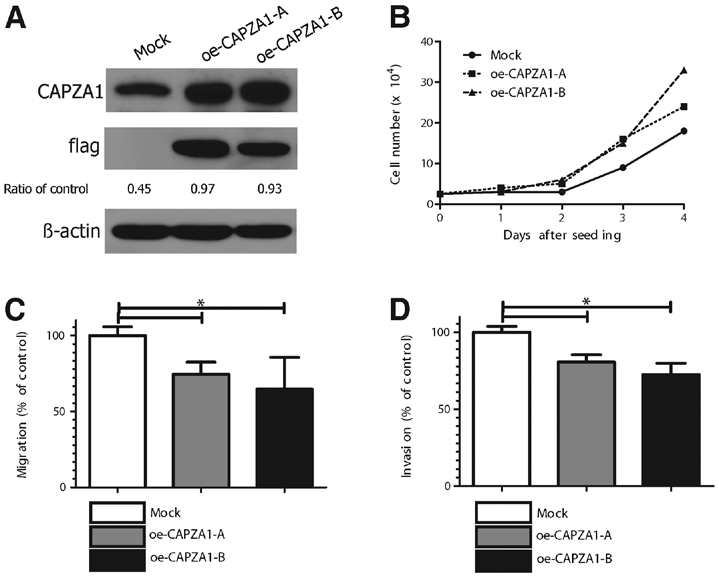
Overexpression of CAPZA1 suppresses gastric cancer cell migration and invasion. (A) Upregulated CAPZA-1 gene expression in three different CAPZA-1-overexpressing MKN45 cells lines. MKN 45 stably transfected with empty (Mock) or CAPZA-1-expressing vector (oe-CAPZA1-A, oe-CAPZA1-B) were analyzed by immunoblotting with an antibody to CAPZA1. An antibody to β-actin was used as an equal loading control. (B) The proliferation rates of the CAPZA1-overexpressing MKN 45 cells were not significantly different from those of control cells. (C) The effect of CAPZA-1 overexpression on *in vitro* migration ability of MKN 45 cells. The migration rates of CAPZA1-overexpressing MKN 45 cells were markedly decreased than that of the control cells. (D) The effect of CAPZA-1 overexpression on *in vitro* invasion ability of MKN 45 cells. The invasion rates of the CAPZA1-overexpressing MKN 45 cells were also markedly decreased compared to that of the control cells. ^*^p<0.01.

The effect of CAPZA1 overexpression on tumor cell proliferation, migration and invasion was assessed in CAPZA1-overexpressing MKN-45 cells. MKN-45 cells stably transfected with empty (mock) or CAPZA-1 expression vector (oe-CAPZA1-A, oe-CAPZA1-B) were analyzed by immunoblotting with an antibody to CAPZA1 (Fig. 5A). The proliferation rate of the CAPZA1-overexpressing MKN-45 cells (oe-CAPZA1-A, oe-CAPZA1-B) was not significantly different from that of control cells (Fig. 5B). The migration rate of CAPZA1-overexpressing MKN-45 cells was markedly decreased compared to that of control cells (p<0.01, Fig. 5C). The invasion rate of the CAPZA1-overexpressing MKN-45 cells was also markedly decreased compared to that of control cells (p<0.01, Fig. 5D).

## Discussion

In our previous proteomic study, CAPZA1 was found to be upregulated in GC tissue compared to normal tissue. As a follow-up, this study was designed to determine whether CAPZA1 may be used as a prognostic marker in GC. Results showed that CAPZA1 overexpression is associated with well differentiated histology, smaller tumor size, higher T1 stage, absence of LN metastasis, lower TNM stage, lower recurrence rate and longer survival. *In vitro* modeling in GC cell lines showed that the overexpression of CAPZA1 markedly suppresses cell migration and invasion and that the depletion of CAPZA1 has the opposite effect. Multivariate analysis of clinicopathological parameters showed that TNM stage is an independent prognostic indicator of cancer-related death. Collectively, these results suggest that CAPZA1 may have prognostic value in gastric cancer. CAPZA1 expression in gastric cancer tissue may help determine treatment choice. For example, in cases of gastric cancer excision by endoscopic submucosal dissection [large T1 mucosal cancer (greater than 2 cm), mixed type of undifferentiated cancer or possibility of submucosal invasion], CAPZA1 expression could be the decisive factor in determining whether surgery should be performed: if CAPZA1 is underexpressed in the cancer tissue, it may be best to recommend surgery over surveillance.

TMAs offer two major advantages: they allow large-scale analysis of human tissues and, through the use of consecutive sections, permit the assessment of multiple proteins in almost all morphologically identical regions of the tumor (23). Recent studies used TMAs to identify biomarkers for breast cancer and brain tumors (24,25), and research has shown that protein expression profiling is clinically useful in the prognostic classification of neoplasms (26). In GC, the expression of EMT-related proteins and the overexpression of EGFR using TMAs were correlated with a poor prognosis and TMA protein expression profiling predicts LN metastasis and prognosis in early stage gastric cancer (7,26,27).

Reports on the role of CAPZA1 in cancer are rare. Differential expression of CAPZA1 has been reported in oral squamous cell carcinoma. A 10-fold increase in the expression of CAPZA1 was observed in HPV18-positive oral squamous cell carcinomas compared to other HPV18-positive cancers, although no overexpression was detected at the RNA level (28). Research has shown that CAPZA2 was amplified in more than 20% of glioblastomas (29). F-actin capping protein has also been implicated in renal cell cancer. This was investigated using PROTEOMEX, an approach that combines conventional proteome analysis with serological screening (30). There is no existing report on the role of CAPZA1 in GC.

Several *in vitro* studies have attempted to elucidate the function of CP. In *Saccharomyces cerevisiae*, disruption of the genes encoding CP results in a disorganized actin cyto-skeleton (31). In *Drosophila melanogaster*, mutations in the CP-b gene affect actin organization, bristle morphology and viability (10). These results indicate that CP is important in cell morphology. CP is also necessary for actin assembly during myofibrillogenesis in cultured muscle cells (32). In a study of CP expression in *Dictylostellum* cells, Hug *et al* found that over-expressing cells moved faster and underexpressing cells moved slower than control cells. The authors also reported that CP mutants exhibited alterations in cytoskeleton architecture (33). Loisel *et al* performed experiments with *Escherichia coli* and *Listeria*, and reported that the rate of cell movement varied with CP concentration and resulted in a bell-shaped curve (34). Initially, the rate of cell movement was fast; however, at very high concentrations, CP blocked the elongation of the actin filaments formed at the bacterium surface, and cell movement became slower. In the present TMA study, 39% of the patients in the CAPZA1 overexpression group had advanced GC (AGC) compared to 60% in the CAPZA1 underexpression group. Among patients with CAPZA1 overexpression, 27% had LN metastasis compared to 41% of those with underexpression. These results suggest that CAPZA1 may stimulate the initial phase of GC development and that a high concentration of CAPZA1 may prevent migration and invasion. Further experiments are needed to investigate this hypothesis. In conclusion, CAPZA1 overexpression may be a suitable marker of good prognosis in GC and is associated *in vitro* with decreased cancer cell migration and invasion.

## Figures and Tables

**Table I:** Clinicopathological data from patients in the tissue microarray experiment.

Pathologic variables	No. of patients
WHO classification	
WD	66
MD	114
PD	99
Mucinous	8
SRC	33
Lauren classification	
Intestinal	181
Diffuse	61
Mixed	7
Tumor size and T stages	
Mean tumor size	4.2±2.6
Stage T1	176
Stage T2	38
Stage T3	185
Stage T4	28
Lymph node metastasis	
Mean no. of involved LN	2.2±5.4
Stage N0	218
Stage N1	36
Stage N2	34
Stage N3	39
TNM stage	
Stage I	198
Stage II	58
Stage III	70
Stage IV	1
Operation	
Subtotal gastrectomy	231
Total	74
Proximal	22
Lymph node dissection	
D1+	115
D2	212
Adjuvant chemotherapy	
No	136
Yes	191
CAPZA1 expression status	
Score: missing value	3
0	56
1	73
2	82
3	63
4	50

Surgically resected gastric cancer tissue specimens were obtained from 327 patients, and medical charts and pathological reports were reviewed to determine clinicopathological parameters. WHO, World Health Organization; WD, well differentiated; MD, moderately differentiated; PD, poorly differentiated; SRC, signet ring cell carcinoma; LN, lymph node; CAPZA1, F-actin capping protein α1 subunit.

**Table II:** Univarate analysis of risk factors for cancer related death in gastric cancer.

	Cancer related death/total patients (%)	Univariate analysis
WHO classification		0.04
WD	4/66 (6.1)	
MD	15/114 (13.2)	
PD	24/99 (24.2)	
Mucinous	1/8 (12.5)	
SRC	4/33 (12.1)	
Pathologic tumor stage		<0.01
T1 (mucosa)	4/176 (2.3)	
T2 (submucosa)	2/38 (5.3)	
T3 (subserosa)	28/85 (32.9)	
T4 (serosa invasion)	15/28 (53.6)	
Pathologic lymph node stage		<0.01
N0	7/218 (3.2)	
N1 (1,2)	6/36 (16.7)	
N2 (3–6)	15/34 (44.1)	
N3 (7-)	21/39 (53.8)	
Pathologic TNM stage		<0.00
I	3/198 (1.5)	
II	9/58 (15.5)	
III–IV	37/71 (52.1)	
Operation		0.09
Subtotal gastrectomy	29/231 (12.6)	
Total gastrectomy	17/74 (23)	
Proximal gastrectomy	3/22 (13.6)	
Lymph node dissection		<0.01
D1+	3/115 (2.6)	
D2	46/212 (21.7)	
Adjuvant chemotherapy		<0.01
No	8/136 (5.9)	
Yes	41/191 (21.5)	
CAPZA1 expression status		0.01
Underexpression (0,1)	28/129 (21.7)	
Overexpression (2–4)	21/195 (10.8)	

WHO, World Health Organization; WD, well differentiated; MD, moderately differentiated; PD, poorly differentiated; SRC, signet ring cell carcinoma; LN, lymph node; CAPZA1, F-actin capping protein α1 subunit.

**Table III:** Comparison of the clinicopathological features in the CAPZA1 underexpression and overexpression groups.

	Levels of CAPZA1 expression		
	Underexpression 0, 1+ (%)	Overexpression 2+, 3+, 4+ (%)	P-value
WHO classification			<0.01
WD	16 (12.6)	49 (25.7)	
MD	44 (34.6)	69 (36.1)	
PD	54 (42.5)	44 (23)	
Mucinous	1 (0.8)	7 (3.7)	
SRC	12 (9.4)	21 (11)	
Lauren classification			0.37
Intestinal	69 (53.5)	110 (56.4)	
Diffuse	30 (23.3)	31 (15.9)	
Mixed	3 (2.3)	4 (2.1)	
Mean tumor size	4.8±2.8	3.7±2.4	<0.01
Pathologic tumor stage			<0.01
T1 (mucosa)	52 (40.3)	121 (62.1)	
T2 (submucosa)	10 (7.8)	28 (14.4)	
T3 (subserosa)	54 (41.9)	31 (15.9)	
T4 (serosa invasion)	13 (10.1)	15 (7.7)	
Pathologic lymph node stage			0.01
N0	73 (56.6)	141 (72.8)	
N1 (1,2)	17 (13.2)	19 (9.7)	
N2 (3–6)	16 (12.4)	18 (9.2)	
N3 (7-)	23 (17.8)	16 (8.2)	
Pathologic TNM stage			<0.01
Stage I	57 (44.2)	138 (70.8)	
Stage II	36 (27.9)	22 (11.3)	
Stages III–IV	36 (27.9)	35 (17.9)	
Lymph node dissection			<0.01
D1+	34 (26.4)	79 (40.5)	
D2	95 (73.6)	116 (59.5)	
Adjuvant chemotherapy	85/129 (65.9)	105/195 (53.8)	0.03
Recurrence	33/129 (25.6)	27/195 (13.8)	<0.01
Cancer related death	28/129 (21.7)	21/195 (10.8)	0.01

WHO, World Health Organization; WD, Well differentiated; MD, moderately differentiated; PD, poorly differentiated; SRC, signet ring cell carcinoma; LN, lymph node; CAPZA1, F-actin capping protein α1 subunit; EGC, early gastric cancer; AGC, advanced gastric cancer; D1+ and D2 is defined Japanese gastric cancer treatment guidelines 2010 (Ver.3).

## References

[1] Lee HJ, Yang HK, Ahn YO (2002). Gastric cancer in Korea. Gastric Cancer.

[2] Kamangar F, Dores GM, Anderson WF (2006). Patterns of cancer incidence, mortality, and prevalence across five continents: defining priorities to reduce cancer disparities in different geographic regions of the world. J Clin Oncol.

[3] Sakuramoto S, Sasako M, Yamaguchi T (2007). Adjuvant chemotherapy for gastric cancer with S-1, an oral fluoropyrimidine. N Engl J Med.

[4] Bang YJ, Van Cutsem E, Feyereislova A (2010). Trastuzumab in combination with chemotherapy versus chemotherapy alone for treatment of HER2-positive advanced gastric or gastrooesophageal junction cancer (ToGA): a phase 3, open-label, randomised controlled trial. Lancet.

[5] Chen CD, Wang CS, Huang YH (2007). Overexpression of CLIC1 in human gastric carcinoma and its clinicopathological significance. Proteomics.

[6] Cho HJ, Baek KE, Park SM (2009). RhoGDI2 expression is associated with tumor growth and malignant progression of gastric cancer. Clin Cancer Res.

[7] Kim MA, Lee HS, Lee HE, Jeon YK, Yang HK, Kim WH (2008). EGFR in gastric carcinomas: prognostic significance of protein overexpression and high gene copy number. Histopathology.

[8] Lim BH, Cho BI, Kim YN, Kim JW, Park ST, Lee CW (2006). Overexpression of nicotinamide N-methyltransferase in gastric cancer tissues and its potential post-translational modification. Exp Mol Med.

[9] Waddle JA, Cooper JA, Waterston RH (1993). The alpha and beta subunits of nematode actin capping protein function in yeast. Mol Biol Cell.

[10] Hopmann R, Cooper JA, Miller KG (1996). Actin organization, bristle morphology, and viability are affected by actin capping protein mutations in Drosophila. J Cell Biol.

[11] Amatruda JF, Cannon JF, Tatchell K, Hug C, Cooper JA (1990). Disruption of the actin cytoskeleton in yeast capping protein mutants. Nature.

[12] Casella JF, Casella SJ, Hollands JA, Caldwell JE, Cooper JA (1989). Isolation and characterization of cDNA encoding the alpha subunit of Cap Z(36/32), an actin-capping protein from the Z line of skeletal muscle. Proc Natl Acad Sci USA.

[13] Barron-Casella EA, Torres MA, Scherer SW, Heng HH, Tsui LC, Casella JF (1995). Sequence analysis and chromosomal localization of human Cap Z. Conserved residues within the actin-binding domain may link Cap Z to gelsolin/severin and profilin protein families. J Biol Chem.

[14] Cooper JA, Caldwell JE, Gattermeir DJ, Torres MA, Amatruda JF, Casella JF (1991). Variant cDNAs encoding proteins similar to the alpha subunit of chicken CapZ. Cell Motil Cytoskeleton.

[15] Tanaka H, Yoshimura Y, Nishina Y, Nozaki M, Nojima H, Nishimune Y (1994). Isolation and characterization of cDNA clones specifically expressed in testicular germ cells. FEBS Lett.

[16] Schafer DA, Korshunova YO, Schroer TA, Cooper JA (1994). Differential localization and sequence analysis of capping protein beta-subunit isoforms of vertebrates. J Cell Biol.

[17] Hart MC, Korshunova YO, Cooper JA (1997). Mapping of the mouse actin capping protein alpha subunit genes and pseudo-genes. Genomics.

[18] Von Bulow M, Rackwitz HR, Zimbelmann R, Franke WW (1997). CP beta3, a novel isoform of an actin-binding protein, is a component of the cytoskeletal calyx of the mammalian sperm head. Exp Cell Res.

[19] Nachmias VT, Golla R, Casella JF, Barron-Casella E (1996). Cap Z, a calcium insensitive capping protein in resting and activated platelets. FEBS Lett.

[20] Barkalow K, Witke W, Kwiatkowski DJ, Hartwig JH (1996). Coordinated regulation of platelet actin filament barbed ends by gelsolin and capping protein. J Cell Biol.

[21] Kimura M, Tsuda H, Morita D (2004). A proposal for diagnostically meaningful criteria to classify increased epidermal growth factor receptor and c-erbB-2 gene copy numbers in gastric carcinoma, based on correlation of fluorescence in situ hybridization and immunohistochemical measurements. Virchows Arch.

[22] Koike N, Todoroki T, Komano H (1997). Invasive potentials of gastric carcinoma cell lines: role of alpha 2 and alpha 6 integrins in invasion. J Cancer Res Clin Oncol.

[23] Lee HS, Kim WH (2006). Tissue array methods for high-throughput clinicopathologic research. Cancer Res Treat.

[24] Ikota H, Kinjo S, Yokoo H, Nakazato Y (2006). Systematic immunohistochemical profiling of 378 brain tumors with 37 antibodies using tissue microarray technology. Acta Neuropathol.

[25] Ou K, Yu K, Kesuma D (2008). Novel breast cancer biomarkers identified by integrative proteomic and gene expression mapping. J Proteome Res.

[26] Lee HS, Cho SB, Lee HE (2007). Protein expression profiling and molecular classification of gastric cancer by the tissue array method. Clin Cancer Res.

[27] Kim MA, Lee HS, Lee HE, Kim JH, Yang HK, Kim WH (2009). Prognostic importance of epithelial-mesenchymal transition-related protein expression in gastric carcinoma. Histopathology.

[28] Lo WY, Lai CC, Hua CH (2007). S100A8 is identified as a biomarker of HPV18-infected oral squamous cell carcinomas by suppression subtraction hybridization, clinical proteomics analysis, and immunohistochemistry staining. J Proteome Res.

[29] Mueller HW, Michel A, Heckel D (1997). Identification of an amplified gene cluster in glioma including two novel amplified genes isolated by exon trapping. Hum Genet.

[30] Kellner R, Lichtenfels R, Atkins D (2002). Targeting of tumor associated antigens in renal cell carcinoma using proteome-based analysis and their clinical significance. Proteomics.

[31] Amatruda JF, Cooper JA (1992). Purification, characterization, and immunofluorescence localization of *Saccharomyces cerevisiae* capping protein. J Cell Biol.

[32] Schafer DA, Hug C, Cooper JA (1995). Inhibition of CapZ during myofibrillogenesis alters assembly of actin filaments. J Cell Biol.

[33] Hug C, Jay PY, Reddy I (1995). Capping protein levels influence actin assembly and cell motility in dictyostelium. Cell.

[34] Loisel TP, Boujemaa R, Pantaloni D, Carlier MF (1999). Reconstitution of actin-based motility of Listeria and Shigella using pure proteins. Nature.

